# Alterations in Vaginal Microbiota Associated with Pregnancy Outcomes in Dairy Cows Revealed by 16S rRNA Gene Sequencing

**DOI:** 10.3390/microorganisms14081718

**Published:** 2026-08-05

**Authors:** Tong Yu, Xiaoge Zhang, Qiuyu Meng, Feng Yang, Yaqi Zhang, Yujie Tang, Xiaoting Zhu, Yangguang Li, Shikun Wang, Zijing Zhang

**Affiliations:** 1 College of Animal Science and Technology, Henan Agricultural University, Zhengzhou 450046, China; 2 College of Veterinary Medicine, Northwest A&F University, Yangling 712100, China; 3 Institute of Animal Husbandry, Henan Academy of Agricultural Sciences, Zhengzhou 450002, China; 4 Henan Huahuaniu Animal Husbandry Technology Co., Ltd., Xinxiang 453500, China13838207621@163.com (S.W.)

**Keywords:** multiparous dairy cows, vaginal microbiota, 16S rRNA gene sequencing, artificial insemination, early embryonic loss, pregnancy status

## Abstract

Early embryonic loss remains a major limitation to reproductive efficiency in dairy cows, with approximately 30–40% of embryonic mortality occurring within 21 days after artificial insemination (AI), ultimately reducing conception rates and economic profitability in dairy production. Increasing evidence suggests that reproductive tract microorganisms may contribute to pregnancy establishment; however, their functions during early gestation in dairy cows remains poorly understood. In this study, 16S rRNA gene amplicon sequencing was used to characterize vaginal microbiota profiles in multiparous Holstein dairy cows at 7.5 and 21 days after AI, with comparisons performed between pregnant and non-pregnant cows. Significant differences in microbial community structure were observed between pregnancy states. At 7.5 days post-AI, distinct differences in microbial composition were detected at the phylum level, with *Bacillota*, *Bacteroidota*, and *Pseudomonadota* identified as dominant phyla. The relative abundance of *Bacillota* was higher in pregnant cows, whereas *Pseudomonadota* was enriched in non-pregnant cows. At the genus level, *Ureaplasma* was significantly increased in pregnant cows, while *Streptococcus* and *Actinobacillus* were significantly enriched in non-pregnant cows. By 21 days after AI, beta diversity analysis revealed a highly significant separation in microbial community structure between pregnant and non-pregnant cows (*p* < 0.01). Volcano plot and LEfSe analyses further identified representative microbial taxa associated with pregnancy status. Notably, inflammatory-associated genera, including *Streptococcus* and *Actinobacillus*, were predominantly enriched in non-pregnant cows, whereas pregnancy-associated taxa, including *Ureaplasma* and *Bifidobacterium*, were enriched in pregnant cows. Collectively, early alterations in the vaginal microbiota after AI were closely associated with pregnancy outcomes. These findings provide insight into the microbial associations related to early pregnancy outcomes and may facilitate the identification of potential microbial biomarkers for predicting pregnancy success in dairy cows.

## 1. Introduction

Reproductive efficiency is a key determinant of productivity and profitability in dairy production systems. In modern intensive dairy farming systems, artificial insemination (AI) is widely applied as the primary reproductive management strategy. However, despite substantial improvements in reproductive management, conception rates in dairy cows remain relatively low, typically ranging from 30% to 40%, and prolonged periods of non-pregnancy continue to be a major challenge [1,2]. Extended days open increase production costs, negatively affect lactation performance, elevate replacement rates, and reduce lifetime productivity, ultimately compromising farm profitability [3]. Therefore, accurate and timely detection of early pregnancy is essential to identify reproductive failure at an early stage and improve overall herd performance.

Numerous studies have demonstrated that pregnancy failure in dairy cows predominantly occurs during early embryonic development, particularly during the period of maternal recognition of pregnancy (MRP) within 21 days after AI. Approximately 30–40% of fertilized embryos are estimated to be lost during this critical period, often without obvious clinical signs, making early embryonic loss difficult to detec [4,5]. Accordingly, focusing on the critical window between 7 and 21 days after AI is essential to identify early indicators of pregnancy failure and to facilitate timely diagnosis and intervention.

Currently, early pregnancy diagnosis in dairy cows primarily relies on measurements of progesterone concentrations in blood or milk, detection of pregnancy-associated glycoproteins (PAGs), and ultrasonographic examination [1]. However, these approaches present limitations when applied before 28 days after AI. Progesterone levels are strongly influenced by luteal function and may result in false-positive diagnoses, whereas PAG concentrations are relatively low during very early gestation, leading to reduced sensitivity and diagnostic accuracy that depends heavily on sampling time [6,7]. In recent years, Doppler color-flow ultrasonography has been developed as an approach for evaluating luteal blood perfusion. This technique enables earlier assessment of pregnancy status at approximately 21 days after mating and exhibits high specificity for identifying non-pregnant cows (up to 99%) with a negative predictive value of 98.1% [8,9,10]. Nevertheless, reliable diagnostic approaches before this time point remain limited. Therefore, identifying novel biological indicators reflecting embryonic development during early pregnancy establishment is of considerable importance.

With the advancement of high-throughput sequencing technologies, increasing attention has been directed toward the role of reproductive tract microbiota in animal reproduction. Accumulating evidence indicates that the vagina and uterus are not sterile environments, and their resident microbial communities may be associated with pregnancy establishment and maintenance through potential links with local immune responses, inflammatory status, and hormonal regulation [11]. In humans and various animal species, dysbiosis of the reproductive tract microbiota has been associated with infertility, pregnancy loss, and abnormal embryonic development [12,13]. In dairy cows, specific pathogenic microorganisms have been associated with reproductive disorders. For example, several pathogenic bacteria have been identified as important causes of pregnancy failure in dairy cows. Infection with *Leptospira interrogans* serovar hardjo has been associated with abortion and reproductive disorders in dairy cows [14,15]. In addition, bovine genital campylobacteriosis caused by *Campylobacter fetus* subspecies is considered a sexually transmitted disease in ruminants, which can lead to reproductive problems such as infertility, early embryonic death, and abortion. These reproductive disorders negatively affect reproductive performance and cause substantial economic losses in livestock production [16]. These findings highlight the potential association between reproductive tract microbiota on reproductive performance. However, in dairy cows, particularly during the critical window of pregnancy establishment within 21 days after AI, systematic investigations examining the association between vaginal microbiota and pregnancy outcomes remain limited [17,18]. Therefore, the present study employed 16S rRNA gene amplicon sequencing to compare the vaginal microbial communities of multiparous Holstein dairy cows at 7.5 and 21 days after AI between pregnant and non-pregnant animals. The objective was to identify microbial signatures associated with early pregnancy outcomes and to elucidate the relationship between vaginal microbiota composition and pregnancy establishment, thereby providing potential microbial candidates for early pregnancy assessment and reproductive management.

## 2. Materials and Methods

### 2.1. Experimental Animals and Management

A total of 27 multiparous Chinese Holstein dairy cows from Henan Huahuaniu Animal Husbandry Technology Co., Ltd. (Xinxiang, China) were selected for this study. Initially, 30 cows were subjected to the same estrus synchronization protocol; however, three cows either returned to estrus or failed to exhibit a normal estrous response during the synchronization period and were therefore excluded from subsequent grouping and analysis. All included cows were clinically healthy, had similar body weights, and showed normal reproductive histories. The parity number ranged from 2 to 4.

All cows were maintained under identical dietary, housing, and management conditions, were housed in the same lactation barn, had ad libitum access to water, and were at comparable lactation stages. Estrus synchronization and artificial insemination were performed within the same period in June. During the experimental period, no antibiotic treatment was administered, and no clinical mastitis or endometritis was observed. Daily management procedures followed the standard operating protocols of the dairy farm.

### 2.2. Reagents and Equipment

A color Doppler veterinary ultrasound system (Zhengzhou Boxianglai Instrument Co., Ltd., Zhengzhou, China) was used for pregnancy diagnosis. Blood samples were collected using vacuum coagulation tubes (KangWeishi Medical Products Co., Ltd., Shijiazhuang, China) and sterile disposable blood collection needles (Aosete Medical-Devices Co., Ltd., Qingdao, China). Vaginal samples were collected using sterile disposable sampling swabs suitable for vaginal sample collection, manufactured by Cida (Guangzhou) Biotechnology Co., Ltd. (Guangzhou, China; medical device registration no. Yuexiezhuzhun 20222221516). The swabs were sterilized by irradiation and individually packaged before use. The swabs consisted of flocked sampling tips suitable for vaginal microbial sample collection. Samples were immediately stored in liquid nitrogen (Tianchi Low Temperature Mechanical Equipment Co., Ltd., Jiaozuo, China) and subsequently transferred to −80 °C until further analysis.

### 2.3. Estrus Synchronization Protocol

A standard estrus synchronization protocol was applied uniformly to all cows. The day of intravaginal progesterone sponge insertion was designated as day 0. At insertion, cows received an intramuscular injection of 100 μg gonadotropin-releasing hormone (GnRH). On day 7, 0.4 mg prostaglandin (PG) was administered intramuscularly, and the intravaginal sponge was removed. On day 9, cows were injected intramuscularly with 100 μg GnRH again. Cows that failed to exhibit normal estrus following synchronization were excluded from subsequent grouping and analysis. Artificial insemination was performed on day 10 based on observed estrous signs, and no additional hormonal treatments were administered during the subsequent sampling period. The semen used for AI was processed using a standard commercial extender containing antibiotics. To minimize potential variation associated with semen-related factors, all cows were inseminated using the same semen batch, insemination protocol, and insemination dose. The day of AI was defined as day 0 post-AI for subsequent sampling and analysis.

### 2.4. Sample Collection and Grouping

Vaginal swab samples were collected on days 7.5 and 21 after AI. Prior to sampling, the external genital area was thoroughly cleaned and disinfected to minimize contamination. Sterile cotton swabs were gently inserted into the vaginal canal to collect secretions. Each swab was immediately transferred into a sterile centrifuge tube, snap-frozen in liquid nitrogen, and stored at −80 °C until DNA extraction and sequencing analysis. Pregnancy status was determined based on subsequent diagnostic examinations. According to the final pregnancy outcomes, samples were categorized into four groups: 7.5-day pregnant (ZRA), 7.5-day non-pregnant (WZRA), 21-day pregnant (ZRB), and 21-day non-pregnant (WZRB).

### 2.5. Pregnancy Determination and Serum Hormone Measurements

Pregnancy status was assessed on days 21 and 33 after AI. At 21 days post-AI, Doppler ultrasonography was performed to assess uterine and luteal blood flow and provide a preliminary assessment of pregnancy status. At 33 days post-AI, transrectal B-mode ultrasonography was conducted to confirm pregnancy by detecting fetal heartbeat or characteristic embryonic structures. Final pregnancy status was determined based on the combined results of both examinations. Cows diagnosed as pregnant at both time points were assigned to the pregnant group, whereas those testing negative at both examinations were classified into the non-pregnant group. Serum concentrations of progesterone (P_4_) and estradiol (E_2_) were measured using bovine-specific ELISA kits (JianCheng Bioengineering Institute, Nanjing, China) according to the manufacturer’s instructions.

### 2.6. DNA Extraction and 16S rRNA Gene Sequencing

In this study, genomic DNA was prepared using the CTAB genomic DNA extraction kit (Novogene, Beijing, China) according to the manufacturer’s instructions. DNA concentration and purity were evaluated as part of routine pre-amplification quality control; however, no predefined numerical thresholds were applied as exclusion criteria. Sample suitability for downstream library construction was primarily determined by successful amplification of the bacterial 16S rRNA V4 region and the quality of polymerase chain reaction (PCR) products for subsequent library preparation. The V4 hypervariable region of the bacterial 16S rRNA gene was amplified by PCR using the universal primers 515F and 806R. Each PCR mixture contained 15 µL of Phusion High-Fidelity PCR Master Mix, 0.2 µM of each primer, and 10 ng of genomic DNA template. PCR amplification was performed with an initial denaturation at 98 °C for 1 min, followed by 30 cycles of denaturation at 98 °C for 10 s, annealing at 50 °C for 30 s, and extension at 72 °C for 30 s, with a final extension at 72 °C for 5 min. PCR products were purified, quantified, and pooled in equimolar concentrations for library construction. Sequencing was performed on an Illumina NovaSeq 6000 platform (Illumina, San Diego, CA, USA) with a read length of 256 bp. The number of raw reads, qualified reads, non-chimeric effective tags, and related quality-control metrics for each sample are provided in Table S1. After quality filtering, denoising, and chimera removal, the minimum number of non-chimeric effective tags retained across all samples was 48,068. To minimize the influence of unequal sequencing depth, all samples were rarefied to 48,068 sequences per sample before alpha and beta diversity analyses. In the present study, formal blank extraction controls were not included during DNA extraction. However, no-template PCR negative controls were included during amplification to monitor potential contamination from PCR reagents and the laboratory environment. Library construction and sequencing were performed only when no detectable amplification was observed in the PCR negative controls.

### 2.7. Bioinformatic and Statistical Analysis

Raw sequencing data were processed using QIIME2 (version 2022.02). After quality filtering and denoising with the DADA2 algorithm integrated within QIIME2, Amplicon sequence variants (ASVs) were generated. Taxonomic classification was performed using the SILVA reference database (version 138.1). Relative abundances of microbial taxa were calculated based on the ASV table and summarized at different taxonomic levels. Alpha diversity indices, including Chao1 and Shannon indices, were calculated to evaluate microbial richness and diversity. Beta diversity was assessed using principal coordinate analysis (PCoA) based on Bray–Curtis distance matrices, and differences among groups were tested using Adonis/PERMANOVA, ANOSIM, or MRPP where appropriate. For Adonis/PERMANOVA analysis, the F statistic, R^2^ value, and *p* value were reported. Confidence intervals were not reported for Adonis/PERMANOVA because this permutation-based distance matrix analysis does not generate confidence intervals in the standard output. Differentially abundant taxa were identified using metagenomeSeq, *t*-test, Kruskal–Wallis test, Wilcoxon test, and LEfSe analysis, depending on the data type and comparison. For metagenomeSeq, *t*-test, Kruskal–Wallis test, and Wilcoxon test, *p* values were adjusted for multiple hypothesis testing using the false discovery rate (FDR) correction, and adjusted *p* values were reported as q values. For Adonis/PERMANOVA, ANOSIM, MRPP, and LEfSe analyses, adjusted q values were not generated; LEfSe results were interpreted using an LDA score threshold of >2.0.

## 3. Results

### 3.1. Pregnancy Diagnosis and Hormonal Assays

**Figure 1:**
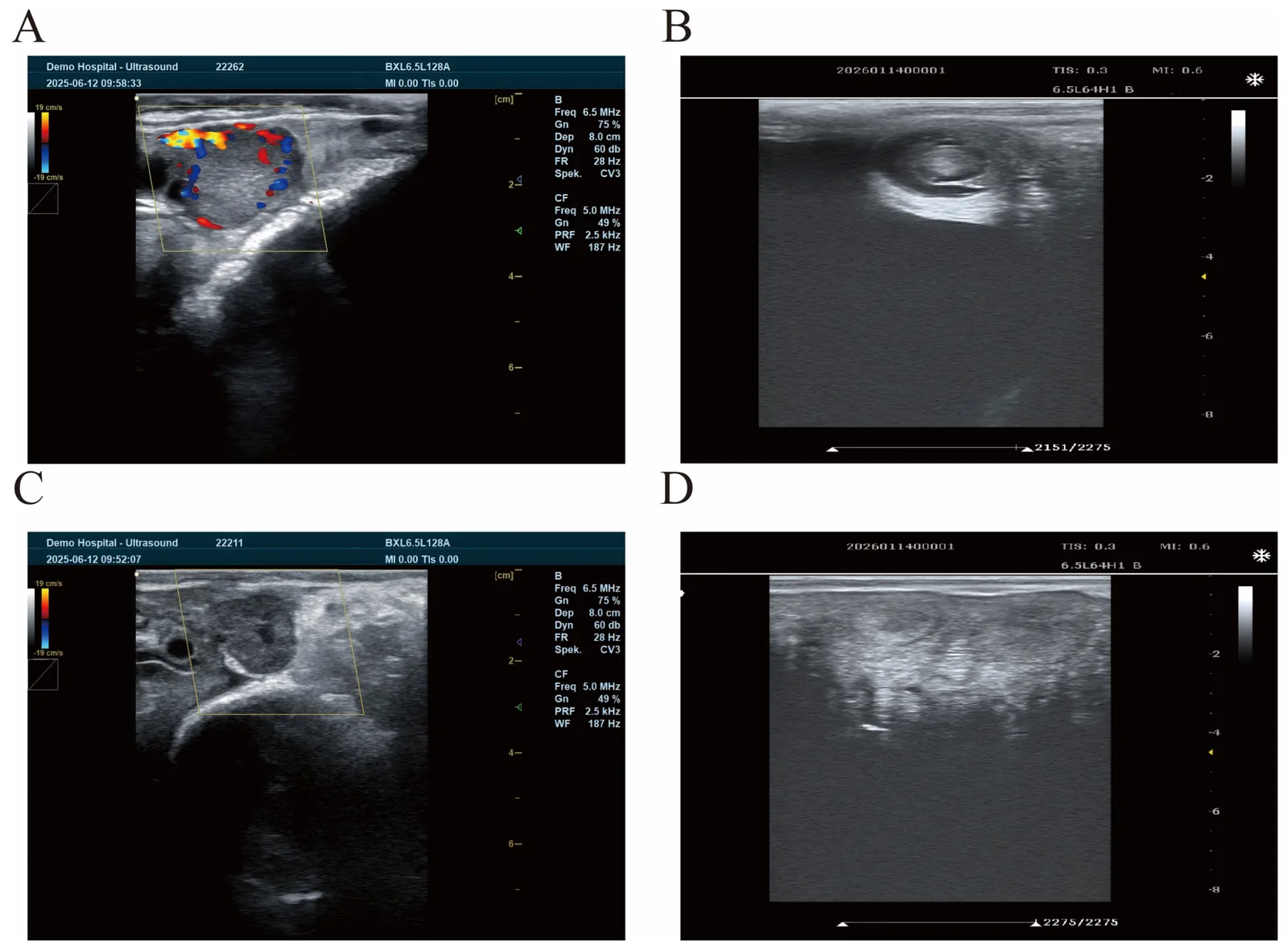
The pregnancy status of Holstein cows at different periods after the application of AI. (**A**) Ultrasound image of a pregnant dairy cow 21 days after AI. (**B**) The B-mode ultrasonography image of a pregnant dairy cow 33 days after AI. (**C**) Ultrasound image of non-pregnant dairy cow 21 days after AI. (**D**) The B-mode ultrasonography image of non-pregnant dairy cow 33 days after AI.

Based on the Doppler color ultrasonography results obtained 21 days after AI, pregnancy status in dairy cows was preliminarily determined according to corpus luteum blood flow. As shown in Figure 1A, cow No. 22262 exhibited abundant blood flow in the corpus luteum, indicating a pregnant status at that time. The B-mode ultrasonography result at 33 days also indicated pregnancy once again (Figure 1B). In contrast, cow No. 22211 showed limited blood flow in the corpus luteum (Figure 1C), suggesting a non-pregnant status. B-mode ultrasonography at 33 days post-AI further confirmed the non-pregnant status (Figure 1D). In total, 14 cows were diagnosed as pregnant and 13 cows were non-pregnant after AI, resulting in a pregnancy rate of 51.85%. Analysis of serum E_2_ and P_4_ concentrations at 7.5 days post-AI revealed no significant differences between pregnant and non-pregnant cows. The E_2_ concentration was 41.290 ± 0.817 pg/mL in pregnant cows and 41.555 ± 0.718 pg/mL in non-pregnant cows (*p* = 0.8101). The P_4_ concentration was 1.454 ± 0.082 ng/mL in pregnant cows and 1.532 ± 0.090 ng/mL in non-pregnant cows (*p* = 0.6867; Supplementary Figure S1).

### 3.2. Species Clustering Patterns and Richness in Pregnant and Non-Pregnant Groups at Different Time Points

Differences in microbial community structures between pregnant and non-pregnant cows at different time points after AI were evaluated using PCoA analysis. The PCoA plots were used to visualize the overall distribution patterns of vaginal microbial communities among groups. To avoid subjective interpretation based only on visual inspection, pairwise Adonis/PERMANOVA analysis was further used to evaluate whether the observed distribution patterns were statistically supported.

**Figure 2:**
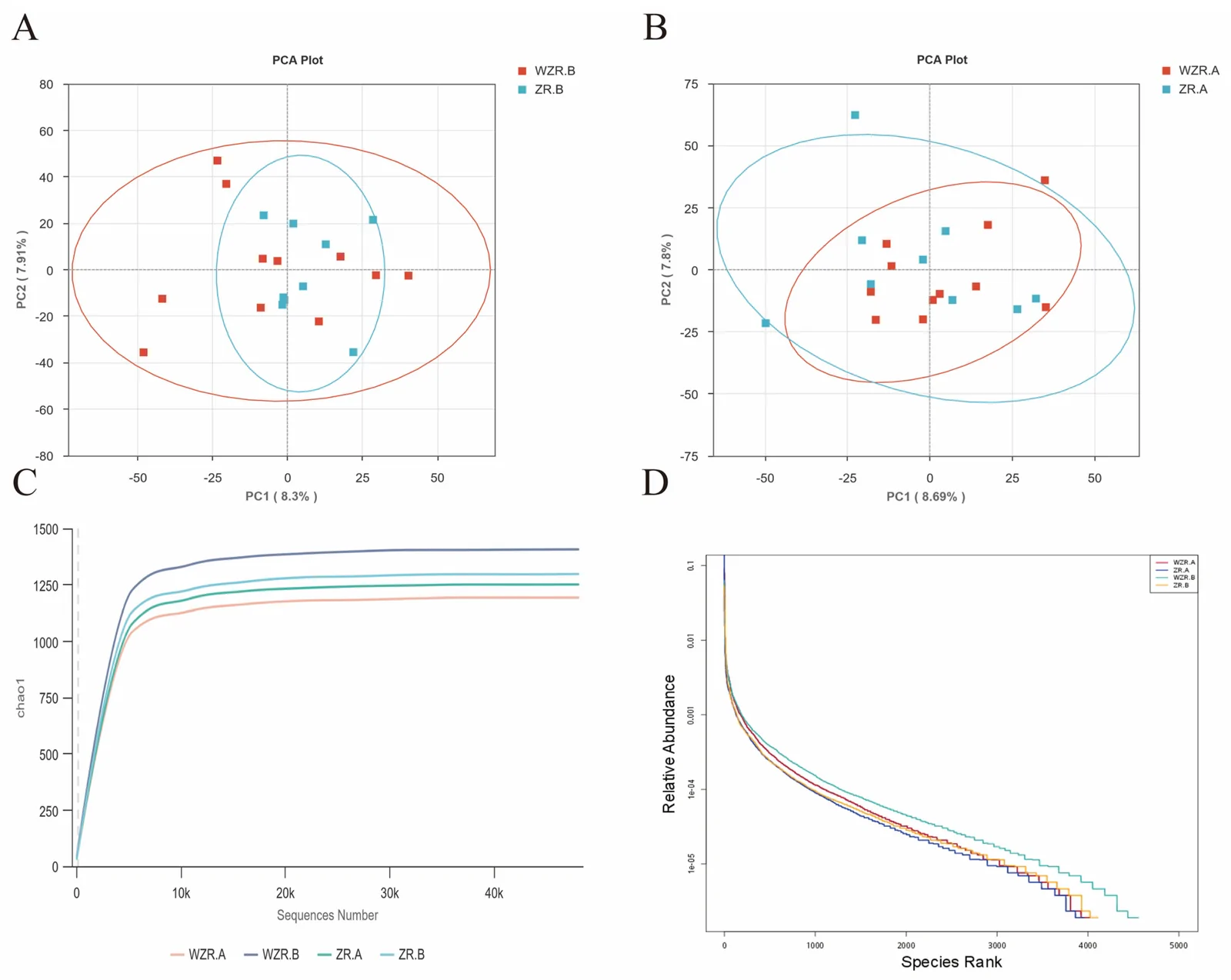
PCOA plots, dilution curves and Rank Abundance curves for the pregnancy and non-pregnancy groups at different times after AI treatment. (**A**) PCOA plot of the WZRB group and the ZRB group. (**B**) PCOA plot of the WZRA group and the ZRA group. (**C**) Dilution curves of the WZRA group, ZRA group, WZRB group, and ZRB group. (**D**) Rank Abundance curves of the WZRA group, ZRA group, WZRB group, and ZRB group.

At 7.5 days after AI, the PCOA plot showed partial overlap between the WZR.A and ZR.A groups, and Adonis analysis indicated that the difference between the two groups was not statistically significant (F = 1.35408, R^2^ = 0.05341, *p* = 0.197, Figure 2). In contrast, at 21 days after AI, the PCoA plot showed a more distinct separation between the WZR.B and ZR.B groups (Figure 2). This separation was supported by Adonis analysis, which showed a significant difference in microbial community structure between the two groups (F = 1.68912, R^2^ = 0.06575, *p* = 0.043). These results suggest that pregnancy-associated differences in vaginal microbial community structure were more evident at 21 days after AI than at 7.5 days after AI.

Rarefaction curves were used to assess whether sequencing depth was sufficient for the samples. The curves of the WZR.A, ZR.A, WZR.B, and ZR.B groups gradually approached a plateau, indicating that the sequencing depth was sufficient to capture most of the microbial diversity in the samples (Figure 2). Rank–abundance curves further showed similar overall patterns among the four groups, suggesting comparable species richness and evenness among samples and their suitability for subsequent analyses (Figure 2).

### 3.3. Composition of Vaginal Microbial Communities in Pregnant and Non-Pregnant Cows at Different Stages

**Figure 3:**
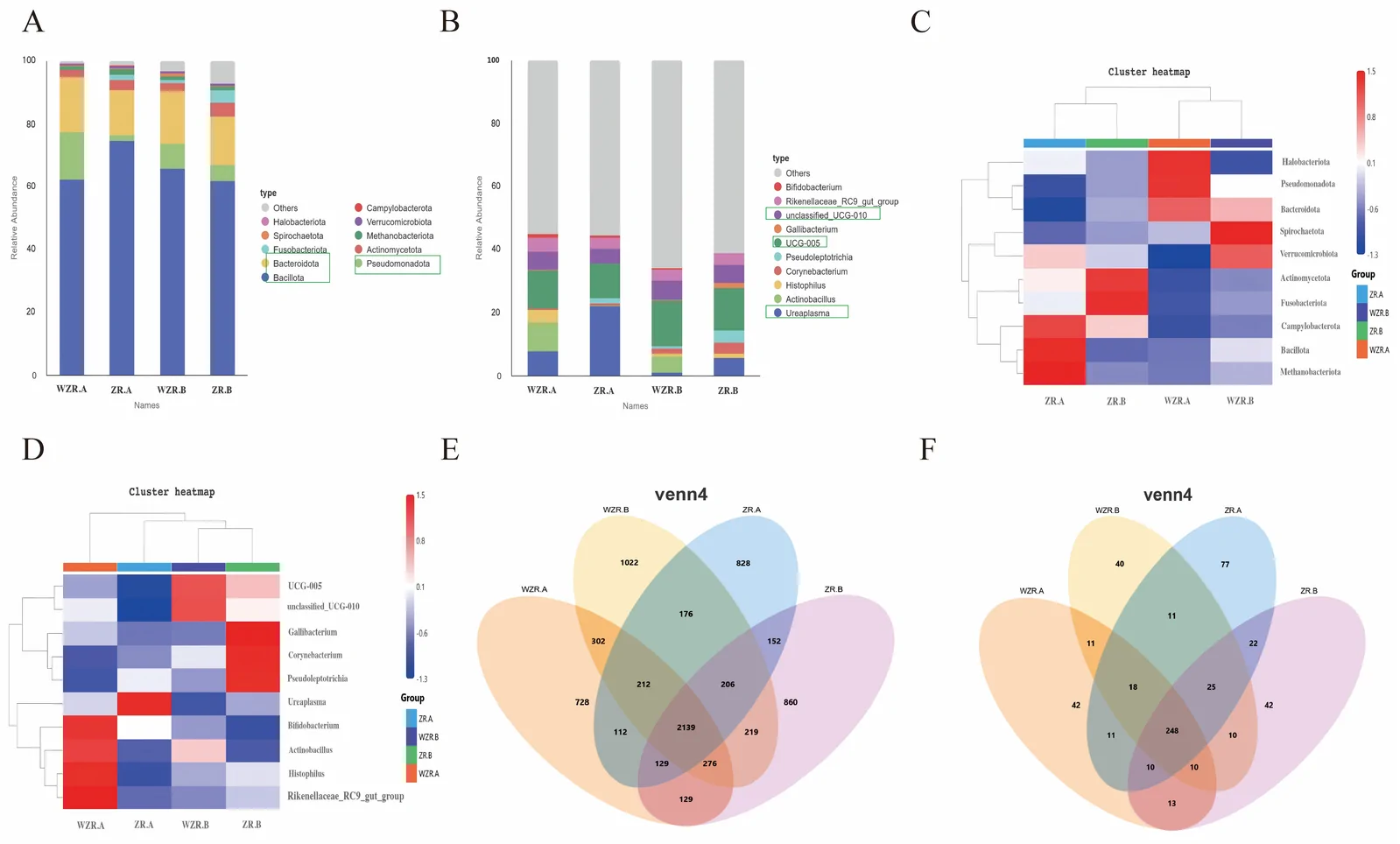
Bar chart of species abundance at the genus and family levels, cluster heatmap and Venn diagram. (**A**) Histogram of species abundance at the phylum level for the WZRA group, ZRA group, WZRB group, and ZRB group. Green frame: key descriptions in high-abundance results. (**B**) Histogram of species abundance at the genus level for the WZRA group, ZRA group, WZRB group, and ZRB group. (**C**) Cluster heatmap at the phylum level for the WZRA group, ZRA group, WZRB group, and ZRB group. (**D**) Cluster heatmap at the genus level for the WZRA group, ZRA group, WZRB group, and ZRB group. (**E**) Species abundance histogram at the genus level for the WZRA group, ZRA group, WZRB group, and ZRB group. (**F**) Microbial species at the ASV level for the WZRA group, ZRA group, WZRB group, and ZRB group.

The vaginal microbial communities of pregnant and non-pregnant cows showed distinct pregnancy-associated compositional patterns at both the phylum and genus levels. Across all groups, Bacillota, Bacteroidota, and Pseudomonadota were the dominant phyla, indicating that these phyla represented the major bacterial components of the bovine vaginal microbiota during the early post-AI period. However, their relative abundances differed between pregnancy outcomes (Figure 3A). At 7.5 days after AI, pregnant cows showed a higher relative abundance of Bacillota and a lower abundance of Pseudomonadota compared with non-pregnant cows, suggesting an early shift in the vaginal microbial environment associated with pregnancy outcome.

At the genus level, *Ureaplasma* showed a pregnancy-associated enrichment pattern. Its relative abundance was higher in pregnant cows than in non-pregnant cows at both 7.5 and 21 days after AI. In contrast, *Actinobacillus* was enriched in non-pregnant cows, whereas its abundance remained relatively low in pregnant cows (Figure 3B). These results indicate that pregnancy outcome was associated with differences in the relative abundances of specific vaginal bacterial taxa during the early post-AI period.

Cluster heatmap and Venn analyses further supported the existence of distinct microbial patterns between pregnancy outcomes. Pregnant cows were characterized by enrichment of pregnancy-associated taxa, including *Ureaplasma* and *Bifidobacterium*, whereas non-pregnant cows showed a broader distribution of taxa associated with microbial imbalance or opportunistic bacterial signatures (Figure 3C–F). However, these taxa should be interpreted as microbial indicators associated with pregnancy status rather than confirmed beneficial, pathogenic, or causal factors.

### 3.4. Overall Microbial Community Structure in Pregnant and Non-Pregnant Cows at Different Time Points

**Figure 4:**
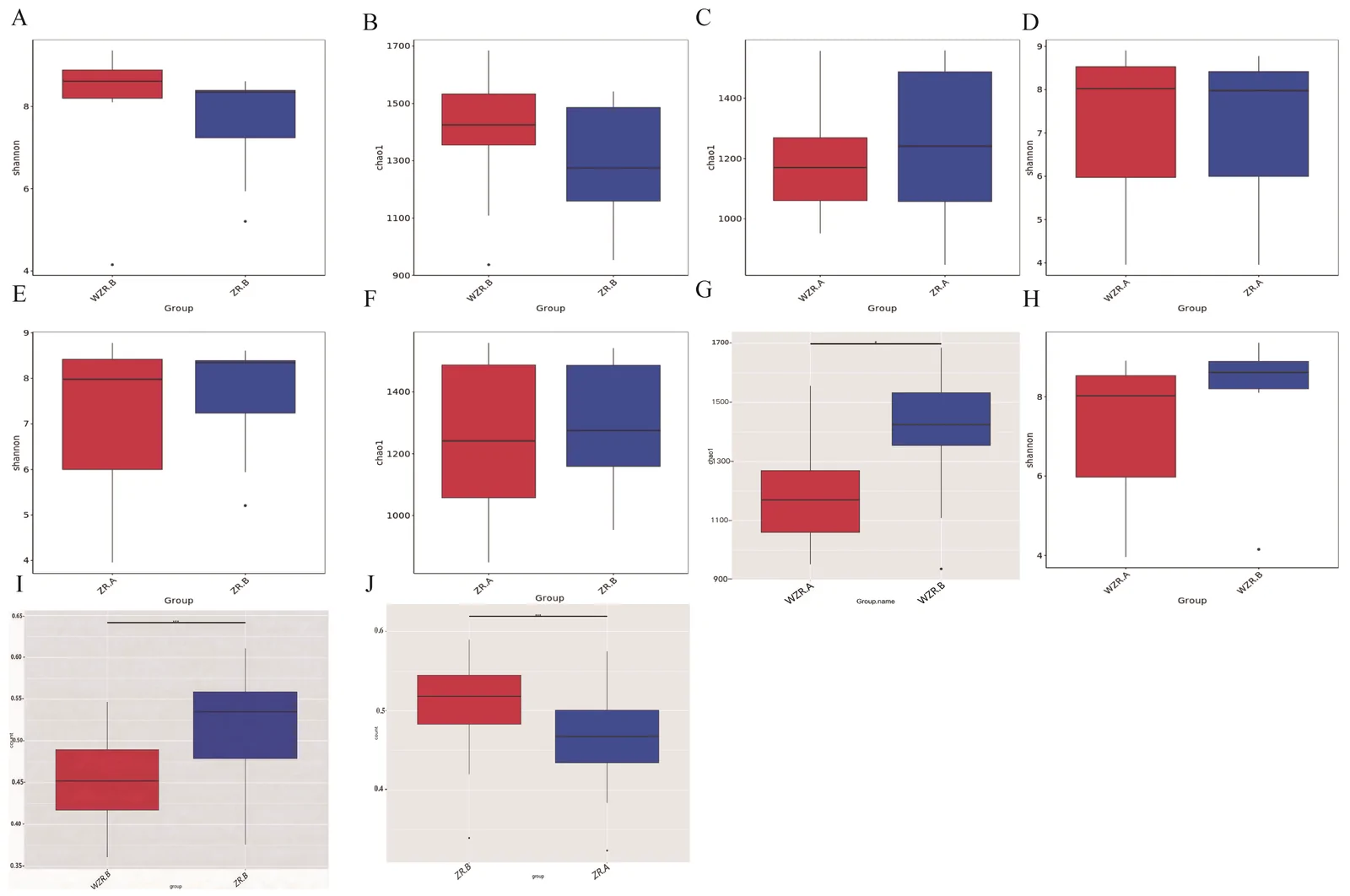
Analysis of differences in Alpha and Beta diversity indices. (**A**) The difference in Shannon index of the Alpha index between the WZRB group and the ZRB group regarding microorganisms. (**B**) The difference in the chao1 index of the Alpha index between the WZRB group and the ZRB group regarding microorganisms. (**C**) The difference in the chao1 index of the Alpha index between the WZRA group and the ZRA group regarding microorganisms. (**D**) The difference in Shannon index of the Alpha index between the WZRA group and the ZRA group regarding microorganisms. (**E**) The difference in Shannon index of the Alpha index between the ZRA group and the ZRB group regarding microorganisms. (**F**) The difference in the chao1 index of the Alpha index between the ZRA group and the ZRB group regarding microorganisms. (**G**) The difference in the chao1 index of the Alpha index between the WZRA group and the WZRB group regarding microorganism. (**H**) The difference in Shannon index of the Alpha index between the WZRA group and the WZRB group regarding microorganisms. (**I**) The difference in Beta index between the WZRB group and the ZRB group regarding microorganisms. (**J**) The difference in Beta index between the ZRA group and the ZRB group regarding microorganisms. (* = *p* < 0.05, *** = *p* < 0.001).

Alpha diversity analysis showed no consistent differences in microbial richness or evenness between pregnant and non-pregnant cows at the same sampling time points. This suggests that pregnancy outcome was not primarily associated with changes in overall microbial richness. Instead, the major difference appeared to lie in the overall structure and composition of the vaginal microbial community (Figure 4A–H).

This observation was further supported by beta diversity analysis. A distinct separation in microbial community structure was observed between pregnant and non-pregnant cows at 21 days after AI (*p* < 0.01), whereas the separation was less evident at 7.5 days after AI (Figure 4I–J). These results suggest that 21 days after AI may represent a key time point when pregnancy-associated microbial differentiation becomes more pronounced. Therefore, the vaginal microbiota associated with pregnancy outcome appears to be characterized mainly by community restructuring rather than by changes in alpha diversity alone.

### 3.5. Differential Microbial Taxa Between Pregnant and Non-Pregnant Groups at Different Time Points

**Figure 5:**
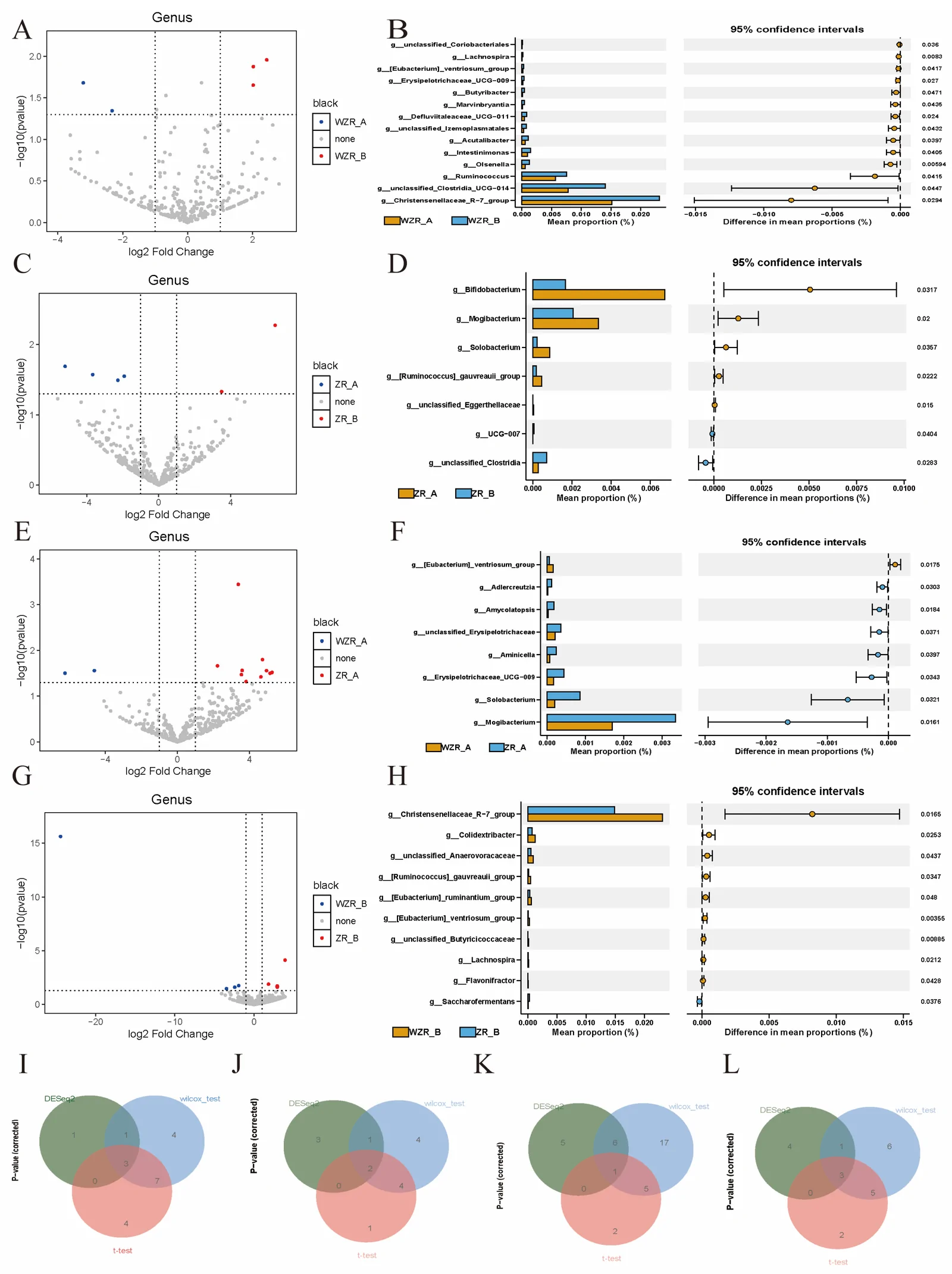
Volcano chart and Venn diagram of pregnancy and non-pregnancy in Holstein dairy cows at different periods after AI. (**A**) Volcano plot of WZRA group and WZRB group. (**B**) Bar chart of KEGG difference results of WZRA group and WZRB group. (**C**) Volcano plot of ZRA group and ZRB group. (**D**) Bar chart of KEGG difference results of ZRA group and ZRB group. (**E**) Volcano plot of WZRA group and ZRA group. (**F**) Bar chart of KEGG difference results of WZRA group and ZRA group. (**G**) Volcano plot of WZRB group and ZRB group. (**H**) Bar chart of KEGG difference results of WZRB group and ZRB group. (**I**) Venn diagram of WZRA group and WZRB group. (**J**) Venn diagram of ZRA group and ZRB group. (**K**) Venn diagram of WZRA group and ZRA group. (**L**) Venn diagram of WZRB group and ZRB group. In the volcano plots, the two vertical dotted lines indicate log2 fold-change cutoffs of −1 and +1, and the horizontal dotted line indicates the significance threshold of *p* = 0.05. Taxa with |log2 fold change| > 1 and *p* < 0.05 were considered significantly different bettween groups.

Differential abundance analysis identified several bacterial taxa that were closely associated with pregnancy status and sampling time. Compared with pregnant cows, non-pregnant cows showed enrichment of several taxa with potential inflammatory or opportunistic pathogenic relevance, including *Actinobacillus*, *Streptococcus*, and *Staphylococcus*. These taxa were particularly evident in comparisons between pregnant and non-pregnant cows, suggesting that the persistence or enrichment of inflammation-associated bacteria may be related to an unfavorable reproductive tract microenvironment (Figure 5).

In contrast, pregnant cows showed enrichment of taxa such as *Bifidobacterium* and *Solobacterium* in specific comparisons. *Bifidobacterium* is often associated with microbial stability and host immune modulation in different mucosal environments, although its specific role in the bovine reproductive tract requires further validation. Therefore, these taxa should be interpreted as pregnancy-associated microbial indicators rather than direct causal (Figure 5).

Comparisons between 7.5 and 21 days after AI further suggested that microbial differences became more structured over time. The non-pregnant group tended to maintain or develop a microbial profile enriched with taxa associated with inflammation or microbial imbalance, whereas the pregnant group showed a more distinct pregnancy-related microbial pattern. These findings suggest that early post-AI microbial dynamics may be associated with pregnancy establishment and early embryonic maintenance.

### 3.6. Representative Differential Microbial Taxa Identified by LEfSe Analysis

**Figure 6:**
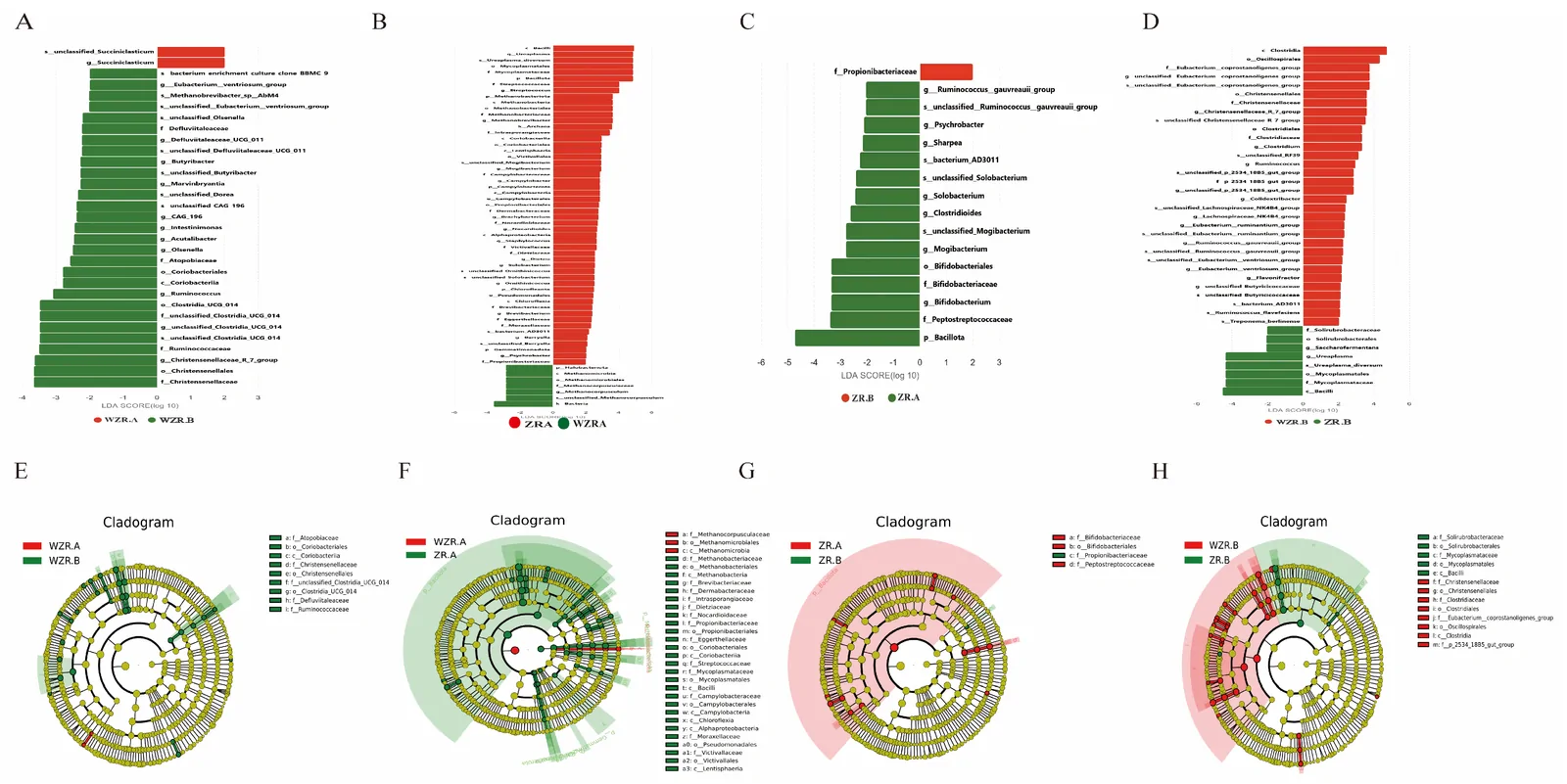
Representative differences in microbial composition between pregnant and non-pregnant Holstein dairy cows at different stages after AI. (**A**) Histogram of LDA values for WZRA group and WZRB group. (**B**) Histogram of LDA values for ZRA group and WZRA group. (**C**) Histogram of LDA values for ZRB group and ZRA group. (**D**) Histogram of LDA values for WZRB group and ZRB group. (**E**) Evolutionary branch diagram of LEfSe for the WZRA group and the WZRB group. (**F**) Evolutionary branch diagram of LEfSe for the ZRA group and the WZRA group. (**G**) Evolutionary branch diagram of LEfSe for the ZRB group and the ZRA group. (**H**) Evolutionary branch diagram of LEfSe for the WZRB group and the ZRB group.

LEfSe analysis further identified representative microbial taxa associated with different pregnancy outcomes. At 7.5 days after AI, *Ureaplasma* and *Streptococcus* were among the most discriminative taxa between pregnant and non-pregnant cows, showing relatively high LDA scores. This finding suggests that microbial differences between pregnancy outcomes may begin early after AI, before conventional pregnancy confirmation (Figure 6).

At 21 days after AI, non-pregnant cows were characterized by the enrichment of taxa such as *Clostridium*, *Ruminococcus*, *Colidextribacter*, and *Flavonifractor*, as well as higher-level taxa including Clostridia and Oscillospirales. Several of these taxa have been reported to be associated with anaerobic metabolism, inflammatory responses, or altered microbial community structure. In contrast, pregnant cows showed enrichment of pregnancy-associated taxa such as *Ureaplasma*, *Bifidobacterium*, *Solobacterium*, and *Propionibacter* in specific comparisons (Figure 6).

Overall, the LEfSe results were consistent with the compositional profiles and beta diversity analyses. The vaginal microbiota of non-pregnant cows tended to be associated with inflammatory or opportunistic bacterial signatures, whereas pregnant cows showed a microbial pattern more closely related to pregnancy establishment or reproductive tract microbial stability. These findings highlight several candidate microbial indicators that may be useful for understanding early pregnancy outcomes in dairy cows.

## 4. Discussion

In the present study, 16S rRNA gene amplicon sequencing was employed to systematically characterize the composition and temporal dynamics of vaginal microbial communities in multiparous Holstein dairy cows at 7.5 and 21 days after AI under different pregnancy statuses. The results indicated that although the dominant bacterial phyla were generally similar between pregnant and non-pregnant cows, the overall vaginal microbial community structure exhibited clear differentiation during the critical period of pregnancy establishment. These findings suggest that reproductive tract microbiota are associated with early pregnancy outcomes in dairy cows, particularly during the early stage of embryonic development following AI.

Alpha diversity analysis showed no significant differences in microbial richness or evenness between pregnant and non-pregnant cows at the same time point (7.5 or 21 days after AI). In contrast, beta diversity analysis revealed a highly significant difference in microbial community structure between pregnant and non-pregnant groups at 21 days post-AI. These findings suggest that pregnancy status was more closely associated with microbial community restructuring and shifts in dominant bacterial taxa than with simple changes in microbial richness alone [19,20]. This observation is consistent with previous studies on vaginal and uterine microbiota of dairy cows, which indicate that pregnancy or disease states are more commonly associated with changes in microbial community composition rather than overall diversity [21].

Previous studies have demonstrated that rapid changes in maternal hormone levels and immune status during early pregnancy can markedly influence the reproductive tract microenvironment [22,23]. For example, studies in humans have shown that elevated estradiol levels promote the maturation of vaginal epithelial cells and increase glycogen deposition. Subsequent enzymatic degradation of glycogen provides a carbon source for *Lactobacillus*, thereby facilitating the establishment and stability of *Lactobacillus*-dominated vaginal microbiota [24,25,26]. *Bifidobacterium* has been associated with mucosal microbial stability and host immune modulation in previous studies, although its specific role in the bovine reproductive tract requires further validation. In contrast, the biological significance of *Ureaplasma* is more complex. In the present study, *Ureaplasma* exhibited a higher relative abundance in pregnant cows and was therefore considered a pregnancy-associated taxon [27,28]. However, this finding does not indicate that *Ureaplasma* is beneficial or causally involved in pregnancy establishment. Importantly, *Ureaplasma diversum* is recognized as an opportunistic bovine pathogen and has been associated with reproductive tract inflammation, reduced fertility, placentitis, abortion, and other reproductive disorders. Therefore, the enrichment of *Ureaplasma* should not be interpreted as inherently beneficial [29,30,31].

Genus-level and LEfSe analysis identified several representative differential bacterial taxa that were closely associated with pregnancy status. In non-pregnant cows, taxa such as *Streptococcus* and *Actinobacillus* were enriched. These genera are considered potential opportunistic bacteria in cattle and may be associated with inflammatory responses or altered mucosal environments under certain host conditions. For instance, Streptococcus and Actinobacteria are frequently identified in bovine mastitis, suggesting that these taxa may play a pathogenic role in inflammation [32]. In conjunction with the higher historical incidence of mastitis or endometritis observed in non-pregnant cows in this study, these findings suggest that systemic or local inflammatory states may alter the reproductive tract microenvironment, thereby adversely affecting embryo implantation and pregnancy maintenance [33,34]. However, the present study did not establish a causal relationship between these taxa and pregnancy failure. Therefore, their enrichment should be interpreted as a microbial signature associated with non-pregnancy under the conditions of this study. In contrast, pregnant cows showed enrichment of taxa such as *Ureaplasma* and *Bifidobacterium*. These taxa may serve as pregnancy-associated microbial indicators but should not be interpreted as confirmed beneficial or causal factors [35]. In particular, the role of *Ureaplasma* requires cautious interpretation because 16S rRNA gene sequencing cannot reliably distinguish species or strains, and some species, such as *Ureaplasma diversum*, have been associated with bovine reproductive disorders [36,37,38]. Further studies using larger cohorts, species-level identification, and quantitative validation are needed to clarify the biological relevance of these differential taxa.

In recent years, the concept of the “gut–reproductive axis” has provided a new perspective for understanding the relationship between microbiota and reproductive performance. Increasing evidence suggests that gut microbiota can influence ovarian function, endometrial receptivity, and pregnancy maintenance through microbial metabolites such as short-chain fatty acids (SCFAs), modulation of inflammatory pathways, and regulation of hormone metabolism, including the estrogen-associated estrobolome [39,40]. Several studies have also indicated that gut microbiota dysbiosis may induce systemic low-grade inflammation and disrupt hormone metabolism, which may indirectly affect the stability of the reproductive tract microbiome [41,42,43]. Consistent with these findings, the enrichment of inflammation-associated bacterial taxa observed in non-pregnant cows in the present study may reflect not only local alterations in the reproductive tract microbiota, but also potential changes in the systemic metabolic and immune status of the host.

It should be noted that the KEGG functional predictions in this study were inferred from 16S rRNA gene amplicon sequencing data using bioinformatic approaches rather than direct metatranscriptomic or metagenomic sequencing. Therefore, the predicted pathways reflect potential functional capacities of the microbial community, rather than actual gene expression or metabolic activity. This distinction should be considered when interpreting the functional implications of the observed microbial alterations. Future studies integrating shotgun metagenomic sequencing or metatranscriptomic analysis are warranted to validate the functional roles of the vaginal microbiota in pregnancy establishment. In addition, this study has limitations related to its single-farm design and the use of 16S rRNA gene sequencing. Moreover, the current study design does not allow discrimination of the independent effects of natural hormonal fluctuations and the AI procedure (including antibiotics present in the semen extender) on vaginal microbial community composition. Although the use of uniform synchronization and AI protocols across all cows minimized inter-individual variation, they also prevented evaluation of the individual contributions of these factors. Therefore, future investigations incorporating additional control groups, such as non-synchronized cycles and non-inseminated cycles, are needed to systematically dissect the relative contributions of these variables to vaginal microbiota dynamics.

In conclusion, this study demonstrated that early alterations in the vaginal microbial community after AI were associated with pregnancy outcomes in dairy cows, with more pronounced differences observed during the critical period of pregnancy establishment (21 days post-AI). Several representative differential microbial taxa identified through LEfSe analysis, including *Ureaplasma*, *Streptococcus*, *Propionibacter*, and *Succiniclasticum*, may serve as potential microbial indicators associated with early pregnancy status. Future studies integrating metagenomic sequencing and multi-omics approaches are warranted to further elucidate the potential relationships between reproductive tract microbiota and early embryonic loss in dairy cows. Such investigations may provide new insights into the development of microbiota-based indicators and management strategies for improving reproductive assessment in dairy cows.

## 5. Conclusions

This study employed 16S rRNA gene amplicon sequencing to characterize the composition and dynamic changes of vaginal microbial communities in dairy cows with different pregnancy outcomes during the early post-AI period. The results indicated that although alpha diversity indices (Chao1 and Shannon) did not differ significantly between pregnant and non-pregnant cows, microbial community structure differed significantly between the two groups. In particular, beta diversity analysis revealed a highly significant difference between the pregnant and non-pregnant groups at 21 days after AI (WZRB vs. ZRB, *p* < 0.01). At the phylum level, the relative abundance of *Bacillota* was higher in the pregnant group at 7.5 days after AI, whereas *Pseudomonadota* showed relatively higher abundance in the non-pregnant group at the same stage. At the genus level, *Ureaplasma* showed highly relative abundance in pregnant cows, whereas *Streptococcus* and *Actinobacillus* were significantly enriched in non-pregnant cows. Overall, the non-pregnant group were characterized by enrichment of taxa potentially associated with inflammatory responses or opportunistic characteristics, such as *Streptococcus* and *Actinobacillus*, whereas the pregnant group showed enrichment of pregnancy-associated taxa, including *Ureaplasma* and *Bifidobacterium*. However, *Ureaplasma* should be interpreted as a genus-level microbial indicator associated with pregnancy status rather than as a confirmed beneficial taxon. These findings indicate that early vaginal microbial community structure is associated with pregnancy outcomes in dairy cows, and that 21 days after AI represents a critical time point for the differentiation of pregnancy-related microbial communities.

## Data Availability

The raw 16S rRNA gene sequencing data generated in this study have been submitted to the NCBI public repository under BioProject accession number PRJNA1492819. According to the NCBI submission information, the data are scheduled to be publicly available on 11 July 2026. Additional data supporting the findings of this study are included in the article and Supplementary Materials.

## Supplementary Materials

The following supporting information can be downloaded at: https://www.mdpi.com/article/10.3390/microorganisms14081718/s1, Figure S1: Serum concentrations of E_2_ and P_4_ in pregnant and non-pregnant dairy cows at 7.5 days after AI. Table S1: Sequencing data quality and filtering statistics.

## References

[1] Fricke P.M., Ricci A., Giordano J.O., Carvalho P.D. (2016). Methods for and Implementation of Pregnancy Diagnosis in Dairy Cows. The Veterinary clinics of North America. Food Anim. Pract..

[2] Walsh S.W., Williams E.J., Evans A.C. (2011). A review of the causes of poor fertility in high milk producing dairy cows. Anim. Reprod. Sci..

[3] De Vries A. (2006). Economic value of pregnancy in dairy cattle. J. Dairy Sci..

[4] Diskin M.G., Morris D.G. (2008). Embryonic and early foetal losses in cattle and other ruminants. Reprod. Domest. Anim. Zuchthyg..

[5] Wiltbank M.C., Baez G.M., Garcia-Guerra A., Toledo M.Z., Monteiro P.L., Melo L.F., Ochoa J.C., Santos J.E., Sartori R. (2016). Pivotal periods for pregnancy loss during the first trimester of gestation in lactating dairy cows. Theriogenology.

[6] Reese S.T., Pereira M.H.C., Edwards J.L., Vasconcelos J.L.M., Pohler K.G. (2018). Pregnancy diagnosis in cattle using pregnancy associated glycoprotein concentration in circulation at day 24 of gestation. Theriogenology.

[7] Ricci A., Carvalho P.D., Amundson M.C., Fourdraine R.H., Vincenti L., Fricke P.M. (2015). Factors associated with pregnancy-associated glycoprotein (PAG) levels in plasma and milk of Holstein cows during early pregnancy and their effect on the accuracy of pregnancy diagnosis. J. Dairy Sci..

[8] Dubuc J., Houle J., Rousseau M., Roy J.P., Buczinski S. (2020). Short communication: Accuracy of corpus luteum color flow Doppler ultrasonography to diagnose nonpregnancy in dairy cows on day 21 after insemination. J. Dairy Sci..

[9] Buzinhani M., Yamaguti M., Oliveira R.C., Cortez B.A., Marques L.M., Machado-Santelli G.M., Assumpção M.E., Timenetsky J. (2011). Invasion of *Ureaplasma diversum* in bovine spermatozoids. BMC Res. Notes.

[10] Crane M.B., Hughes C.A. (2018). Can *Ureaplasma diversum* be transmitted from donor to recipient through the embryo? Two case reports outlining *U. diversum* losses in bovine embryo pregnancies. Can. Vet. J..

[11] Sheldon I.M., Cronin J.G., Bromfield J.J. (2019). Tolerance and Innate Immunity Shape the Development of Postpartum Uterine Disease and the Impact of Endometritis in Dairy Cattle. Annu. Rev. Anim. Biosci..

[12] Fettweis J.M., Serrano M.G., Brooks J.P., Edwards D.J., Girerd P.H., Parikh H.I., Huang B., Arodz T.J., Edupuganti L., Glascock A.L. (2019). The vaginal microbiome and preterm birth. Nat. Med..

[13] Franasiak J.M., Scott R.T. (2015). Reproductive tract microbiome in assisted reproductive technologies. Fertil. Steril..

[14] Grippi F., Giudice E., Pietro S.D., Sciacca C., Santangelo F., Galluzzo P., Barreca S., Guercio A. (2020). *Leptospira interrogans* Serogroup Sejroe Serovar Hardjo in Aborting Cows: Two Herd Cases in Sicily (Italy). J. Vet. Res..

[15] Te Brugge L.A., Dreyer T. (1985). *Leptospira interrogans* serovar hardjo associated with bovine abortion in South Africa. Onderstepoort J. Vet. Res..

[16] Silveira C.D.S., Fraga M., Giannitti F., Macías-Rioseco M., Riet-Correa F. (2018). Diagnosis of Bovine Genital Campylobacteriosis in South America. Front. Vet. Sci..

[17] Ault T.B., Clemmons B.A., Reese S.T., Dantas F.G., Franco G.A., Smith T.P.L., Edwards J.L., Myer P.R., Pohler K.G. (2019). Uterine and vaginal bacterial community diversity prior to artificial insemination between pregnant and nonpregnant postpartum cows1. J. Anim. Sci..

[18] Moraes J.G.N., Gull T., Ericsson A.C., Poock S.E., Caldeira M.O., Lucy M.C. (2024). The microbiome of the pregnant uterus in Holstein dairy heifers and cows assessed by bacterial culture and 16S ribosomal RNA gene sequencing. Front. Microbiol..

[19] Calleros L., Barcellos M., Grecco S., Garzón J.P., Lozano J., Urioste V., Gastal G. (2024). Longitudinal study of the bovine cervico-vaginal bacterial microbiota throughout pregnancy using 16S ribosomal RNA gene sequences. Infect. Genet. Evol. J. Mol. Epidemiol. Evol. Genet. Infect. Dis..

[20] Wang Y., Ametaj B.N., Ambrose D.J., Gänzle M.G. (2013). Characterisation of the bacterial microbiota of the vagina of dairy cows and isolation of pediocin-producing *Pediococcus acidilactici*. BMC Microbiol..

[21] Ravel J., Gajer P., Abdo Z., Schneider G.M., Koenig S.S., McCulle S.L., Karlebach S., Gorle R., Russell J., Tacket C.O. (2011). Vaginal microbiome of reproductive-age women. Proc. Natl. Acad. Sci. USA.

[22] Mirmonsef P., Hotton A.L., Gilbert D., Gioia C.J., Maric D., Hope T.J., Landay A.L., Spear G.T. (2016). Glycogen Levels in Undiluted Genital Fluid and Their Relationship to Vaginal pH, Estrogen, and Progesterone. PLoS ONE.

[23] Robertson S.A., Care A.S., Moldenhauer L.M. (2018). Regulatory T cells in embryo implantation and the immune response to pregnancy. J. Clin. Investig..

[24] Mirmonsef P., Modur S., Burgad D., Gilbert D., Golub E.T., French A.L., McCotter K., Landay A.L., Spear G.T. (2015). Exploratory comparison of vaginal glycogen and Lactobacillus levels in premenopausal and postmenopausal women. Menopause.

[25] Amabebe E., Anumba D.O.C. (2018). The Vaginal Microenvironment: The Physiologic Role of Lactobacilli. Front. Med..

[26] Spear G.T., French A.L., Gilbert D., Zariffard M.R., Mirmonsef P., Sullivan T.H., Spear W.W., Landay A., Micci S., Lee B.H. (2014). Human α-amylase present in lower-genital-tract mucosal fluid processes glycogen to support vaginal colonization by Lactobacillus. J. Infect. Dis..

[27] Wang R., Zhou G., Wu L., Huang X., Li Y., Luo B., Zhu H., Huang W. (2021). The Microbial Composition of Lower Genital Tract May Affect the Outcome of in vitro Fertilization-Embryo Transfer. Front. Microbiol..

[28] Freitas A.C., Chaban B., Bocking A., Rocco M., Yang S., Hill J.E., Money D.M. (2017). The vaginal microbiome of pregnant women is less rich and diverse, with lower prevalence of Mollicutes, compared to non-pregnant women. Sci. Rep..

[29] Oh K.Y., Lee S., Park J., Park M.H., Jeong J.H., Yang J.B., Lim C.K., Ha J.G., Yang Y.S. (2024). Vaginal microbiota of pregnant women with *Ureaplasma urealyticum* and *Mycoplasma hominis* infections. Front. Cell. Infect. Microbiol..

[30] Santos Junior M.N., de Macêdo Neres N.S., Campos G.B., Bastos B.L., Timenetsky J., Marques L.M. (2021). A Review of *Ureaplasma diversum*: A Representative of the Mollicute Class Associated with Reproductive and Respiratory Disorders in Cattle. Front. Vet. Sci..

[31] Waites K.B., Katz B., Schelonka R.L. (2005). Mycoplasmas and ureaplasmas as neonatal pathogens. Clin. Microbiol. Rev..

[32] Poole R.K., Soffa D.R., McAnally B.E., Smith M.S., Hickman-Brown K.J., Stockland E.L. (2023). Reproductive Microbiomes in Domestic Livestock: Insights Utilizing 16S rRNA Gene Amplicon Community Sequencing. Animals.

[33] Miller R., Chelmonska-Soyta A., Smits B., Foster R., Rosendal S. (1994). *Ureaplasma diversum* as a cause of reproductive disease in cattle. Vet. Clin. N. Am. Food Anim. Pract..

[34] Cardoso M.V., Scarcelli E., Grasso L.M., Teixeira S.R., Genovez M.E. (2000). *Ureaplasma diversum* and reproductive disorder in Brazilian cows and heifers; first report. Anim. Reprod. Sci..

[35] Smith M.S., Hickman-Brown K.J., McAnally B.E., Oliveira Filho R.V., de Melo G.D., Pohler K.G., Poole R.K. (2023). Reproductive microbiome and cytokine profiles associated with fertility outcomes of postpartum beef cows. J. Anim. Sci..

[36] Kudo H., Sugiura T., Higashi S., Oka K., Takahashi M., Kamiya S., Tamura Y., Usui M. (2021). Characterization of Reproductive Microbiota of Primiparous Cows During Early Postpartum Periods in the Presence and Absence of Endometritis. Front. Vet. Sci..

[37] Díaz J.M., Prieto A., López G., Díaz P., López C., Quintela L., Morrondo P., Fernández G. (2019). Association of *Ureaplasma diversum* with reproductive disease in cattle. N. Z. Vet. J..

[38] Adnane M., Chapwanya A. (2024). Microbial Gatekeepers of Fertility in the Female Reproductive Microbiome of Cattle. Int. J. Mol. Sci..

[39] Ashonibare V.J., Akorede B.A., Ashonibare P.J., Akhigbe T.M., Akhigbe R.E. (2024). Gut microbiota-gonadal axis: The impact of gut microbiota on reproductive functions. Front. Immunol..

[40] Li B., Xiong Y., Guo D., Deng G., Wu H. (2025). The gut-reproductive axis: Bridging microbiota balances to reproductive health and fetal development. Int. Immunopharmacol..

[41] Moustakli E., Stavros S., Katopodis P., Potiris A., Drakakis P., Dafopoulos S., Zachariou A., Dafopoulos K., Zikopoulos K., Zikopoulos A. (2025). Gut Microbiome Dysbiosis and Its Impact on Reproductive Health: Mechanisms and Clinical Applications. Metabolites.

[42] Qi X., Yun C., Pang Y., Qiao J. (2021). The impact of the gut microbiota on the reproductive and metabolic endocrine system. Gut Microbes.

[43] Yao X., Dong S., Guan W., Fu L., Li G., Wang Z., Jiao J., Wang X. (2023). Gut Microbiota-Derived Short Chain Fatty Acids Are Associated with Clinical Pregnancy Outcome in Women Undergoing IVF/ICSI-ET: A Retrospective Study. Nutrients.

