# Supplementary material for: Alterations in Vaginal Microbiota Associated with Pregnancy Outcomes in Dairy Cows Revealed by 16S rRNA Gene Sequencing
Source: Microorganisms. 2026 Aug 5;14(8):1718. doi: 10.3390/microorganisms14081718 (PMC13515298; doi:10.3390/microorganisms14081718)
Supplement: Supplementary file 1 [file microorganisms-14-01718-s001.zip › Supplementary Figure.pdf]

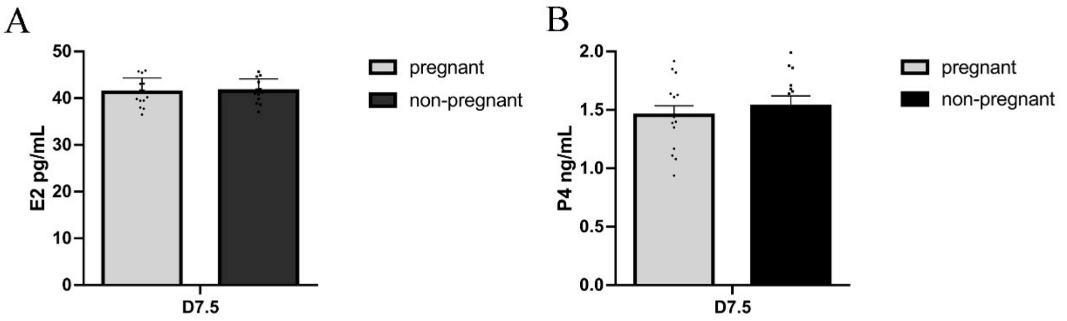

Supplementary Figure S1. Serum concentrations of E_2 and P_4 in pregnant and non-pregnant dairy cows at 7.5 days after AI. (A) Serum E_2 concentrations in pregnant and non-pregnant cows. (B) Serum P_4 concentrations in pregnant and non-pregnant cows. Serum E_2 and P_4 concentrations were measured using bovine-specific ELISA kits. No significant differences were observed in serum E_2 or P_4 concentrations between pregnant and non-pregnant cows at 7.5 days after AI. Data are presented as mean ± SEM. Statistical differences between groups were analyzed using an independent samples t-test.
